# Perfluorooctanoic acid in indoor particulate matter triggers oxidative stress and inflammation in corneal and retinal cells

**DOI:** 10.1038/s41598-020-72600-8

**Published:** 2020-09-24

**Authors:** Peng-Tai Tien, Hui-Ju Lin, Yi-Yu Tsai, Yun-Ping Lim, Chih Sheng Chen, Ching-Yao Chang, Chao-Jen Lin, Jamie Jiin-Yi Chen, Shan-Mei Wu, Yuh-Jeen Huang, Lei Wan

**Affiliations:** 1 Graduate Institute of Clinical Medical Science, College of Medicine, China Medical University, Taichung, Taiwan; 2 Department of Ophthalmology, China Medical University Hospital, Taichung, Taiwan; 3 School of Chinese Medicine, China Medical University, No. 91, Hsueh-Shih Road, Taichung, 40402 Taiwan; 4 Department of Pharmacy, College of Pharmacy, China Medical University, Taichung, Taiwan; 5 Division of Chinese Medicine, Asia University Hospital, Taichung, Taiwan; 6 Department of Biotechnology, Asia University, Taichung, Taiwan; 7 Department of Pediatrics, Changhua Christian Children’s Hospital, Changhua, Taiwan; 8 School of Medicine, Chung Shan Medical University, Taichung, Taiwan; 9 Department of Biomedical Engineering and Environmental Sciences, National Tsing Hua University, 101, Section 2, Kuang-Fu Road, Hsinchu, 30013 Taiwan; 10 Institute of Analytical and Environmental Sciences, National Tsing Hua University, Hsinchu, Taiwan; 11 Department of Obstetrics and Gynecology, China Medical University Hospital, Taichung, Taiwan

**Keywords:** Environmental impact, Macular degeneration

## Abstract

To investigate the particle size distribution of particulate matter and the concentration of specific perfluorinated compounds in indoor dust samples from several locations. Then, we used cell-based assays to investigate the effect of perfluorinated compounds on human corneal epithelial (HCEpiC), endothelial cells (HCEC) and retinal pigment epithelial cells (RPE). Indoor dust samples were collected at five different locations and PM_50–10_, PM_10–2.5_, and PM_2.5–1_ were fractionized. The presence and levels of 8:2 fluorotelomer alcohol, 10:2 fluorotelomer alcohol, and perfluorooctanoic acid were detected by gas chromatography–mass spectrometry. The effect of perfluorooctanoic acid on the activation of reactive oxygen species, transepithelial resistance as well as the expression of interleukin (IL)-6 and IL-8 were determined. The basolateral media of human corneal epithelial or human corneal endothelial cells were used to treat human corneal endothelial or retinal pigment epithelial cells, respectively to indicate the potential of ocular surface inflammation may result in retinal inflammation. Among perfluorinated compounds, only perfluorooctanoic acid was detected in all indoor dust samples. Perfluorooctanoic acid had the highest concentration among all perfluorinated compounds in the samples. Exposure to perfluorooctanoic acid impaired tight junction sealing and increased the levels of reactive oxygen species in human corneal epithelial cells. In human corneal epithelial cells, secretion of IL-6 and IL-8 in both apical and basolateral media was promoted significantly by perfluorooctanoic acid treatment. Stimulation with the basolateral media from perfluorooctanoic acid-treated human corneal epithelial cells induced inflammation in human corneal endothelial cells. The treatment of retinal pigment epithelial cells with the basolateral media from stimulated human corneal endothelial cells also elicited the secretion of proinflammatory cytokines. The results indicate that perfluorooctanoic acid exposure impaired the tight junction of corneal cells and caused inflammatory reactions in the retina. Exposure of the cornea to perfluorooctanoic acid contained in particulate matter might induce oxidative stress and inflammation in the retina and represent a risk factor for age-related macular degeneration.

## Introduction

Air pollution derived from human activities seems to be inevitable and it is the most widespread type of environmental pollution. Diverse human activities generate particulate matter (PM), a general term that refers to a myriad of suspended solid particles and liquid droplets varying in size and composition. The levels of PM in indoor environments may exceed those found outdoors, with activities such as cooking and smoking largely accounting for the accumulation of PM in domestic homes^1–3^. Since people spend a great part of their lives indoors, indoor PM (IPM) may play important roles in altering human health: specific activities, such as cleaning by sweeping and vacuuming the floor or carpets, result in PM resuspension and thus acute exposure to high concentrations of PM. During housekeeping activities, particles smaller than 2.5 µm in diameter (PM_2.5–1_) are often resuspended and, owing to their small size, they can penetrate deeper into the lung and alveoli. Thus, PM_2.5–1_ are more strongly associated with detrimental health effects than larger PM.

Most studies so far have confirmed that exposure to small PM, such as PM_2.5_, is associated with several short- and long-term health problems including lung and heart diseases, the development of several types of cancer, as well as increased mortality and premature death^4–8^; only few reports address the harmful effects of PM on the eyes, a delicate organ that, given its anatomical location, is extremely vulnerable to the effects of air pollution. In fact, in areas with high levels of air pollution, the number of outpatient visits because of non-specific conjunctivitis has increased^9–12^. Exposure to PM_2.5_ in particular causes eye irritation, burning sensation, and allergic eye diseases, which are accompanied by dry and inflamed eyes^13^. The outermost layers of the eye, the conjunctiva and the cornea, are in direct contact with the outside environment^14^ and are more vulnerable to suspended particles in the air. Specifically, exposure to PM_2.5_ limits the corneal wound healing capacity by inhibiting the migration of corneal epithelial cells to the damaged areas^14,15^. Moreover, PM_2.5_ enhances the production of reactive oxygen species (ROS) in the cornea and promotes inflammatory reactions and apoptosis of corneal epithelial cells, leading to senescence of these cells^16^. In addition, exposure to PM_2.5_ is associated with low levels of the anti-inflammatory cytokine interleukin (IL)-10 in the tears of healthy outdoor workers^11^.

Perfluorinated compounds (PFCs) compose a family of man-made chemicals commonly found in PM and IPM. These compounds are widely used as a component of the coatings for raincoats, carpets, popcorn bags, and non-stick cookware because of their high thermal stability and low adhesion feature. It has been shown that indoor levels of PFCs are 2- to 4-fold higher than those outdoors because of the limited ventilation in those relatively closed environments^17^. The presence of lipophobic/hydrophobic perfluoroalkyl tails with charged and polar head groups in PFCs allows these chemicals to interact with biological membranes and proteins^18^, to disturb cell–cell communication, and to impair mitochondrial function^19,20^. These may lead to adverse outcomes, since PFCs are able to enter the circulation. It was reported previously that males tend to have higher PFC serum concentration than females among non-Hispanic white, non-Hispanic black, and Mexican American populations, irrespective of the age group^21^. In addition, PFCs disrupt endocrine functions and, at high concentration, they can decrease the semen quality^22^. Moreover, PFCs are able to cross the placental barrier and alter the normal development of embryos and fetuses^23^. In addition to the deleterious effects of circulating PFCs, it is known that these compounds also affect tissues directly exposed to the environment, such as the respiratory tract surface, skin, and eyes.

Age-related macular degeneration (AMD) is an eye disease that is the primary cause of permanent central blindness in the elderly living in developed countries. Advanced aging, genetic factors, and environmental stress are risk factors for AMD. At the cellular level, altered biological activity of the retinal pigment epithelium (RPE) is one of the key pathophysiological features of AMD. These cells are considered specialized stationary macrophages, which are associated with shedding and clearance of the posterior outer segment of photoreceptors to maintain visual acuity. However, the ability of RPE to digest and remove photoreceptors decreases with aging and induces the accumulation of covalent cross-linked protein aggregates known as lipofuscin. Lipofuscin activates the intracellular production of reactive oxygen species (ROS), which further damages RPE cells. Among other molecules, N-retinylidene-N-retinylethanolamine (A2E) is the major component of lipofuscin from RPE, which is a by-product of the visual cycle^24^. Studies have shown that A2E is the main component of lipofuscin and can damage RPE cell through initiating lysosomal membrane disintegration, induce ROS generation, and promote AMD^25^.

To better understand the effects of particulate matter on the macular degeneration process, we first analyzed the particle size distribution and the concentration of specific PFCs in indoor dust samples from several locations. Then, we used cell-based assays to assess whether perfluorooctanoic acid (PFOA), a common PFC, affects human corneal epithelial and endothelial cells, as well as retinal pigment epithelial cells. We hypothesized that PFOA may directly affect these cells to initiate biological processes that lead to degenerative reactions in the eye, and thus represent a risk factor for the development of AMD.

## Results

### Perfluorinated compounds concentrated in PM_2.5_

After sieving PM from the five locations through a 50-µm filter, the contribution of each particle size range to the total weight of the dust samples were 90.7–95.81%, 3.64–7.33%, 0.25–1.90%, and 0.01–0.07% for PM_50–10_, PM_10–2.5_, PM_2.5–1_, and PM_<1_, respectively (Supplementary Table 2). The different indoor environments did not have any significant effect on the relative contribution of particles separated by size to the weight of the dust. The concentrations of PFCs were inversely correlated with the size of particles (Table 1). The concentrations of 8:2 FTOH, 10:2 FTOH, and PFOA were approximately 1.5-fold and tenfold higher in PM_2.5–1_ than in PM_10–2.5_ and PM_50–10_, respectively. However, for larger particles, each particle could contain higher amount of perfluorinated compounds than the smaller one. Furthermore, the perfluorinated concentrations were similar when the unit was according to particle surface area. Through the information, the higher mass concentration of PFCs in PM_2.5–1_ could be explained by the higher particle number and total surface area than the other size ranges (Supplementary Table 3). Since the smallest particles (PM_2.5–1_) are known to be strongly correlated with detrimental health effects and presented the highest concentrations of PFCs, we chose this fraction to perform further experiments.

**Table 1:** Concentrations of perfluorinated compounds among different sized particles collected in library#1.

Particle size (μm)	Concentration (ng/g), RSD (%)					
	8:2 FTOH		10:2 FTOH		PFOA	
10–50	2763	8.7	995	7.5	2320	8.7
2.5–10	14,542	7.4	6262	6.7	17,088	4.2
1–2.5	24,796	3.2	10,515	5.5	26,882	3.9

*RSD* relative standard deviation.

### Perfluorooctanoic acid is widely found in different environments

The concentration of PFCs in the PM_2.5–1_ fractions collected from different places were determined. Only library 1 had detectable levels of 8:2 FTOH and 10:2 FTOH, whereas PFOA was detected in dust samples from all locations (Supplementary Table 4). The carpets in library 1 and 2 are from the same manufacturer; the carpet in library 1 had been used for three years, whereas the carpet in library 2 was more than 20 years old. Comparing the new carpet with the 3-year-old carpet (library 1) and with the 20-year-old carpet (library 2), we found that the concentrations of PFCs correlated with age (Table 2). As shown in Table 2, the concentrations of 8:2 FTOH, 10:2 FTOH, and PFOA were the highest in the new carpet and gradually decreased over time. The levels of 8:2 FTOH and 10:2 FTOH were not detectable in the 20-year-old carpet.

**Table 2:** Concentrations of perfluorinated compounds in the carpet at different age.

Compound	Concentration (ng/g), RSD (%)					
	New		3 years		20 years	
8:2 FTOH	21,416	6.0	9638	3.0	ND	
10:2 FTOH	11,792	3.0	3820	6.0	ND	
PFOA	37,458	1.3	10,339	7.5	9486	6.3

*ND*: not detectable.

In the accelerated aging test, the concentrations of PFCs in the carpets were reduced by approximately 20% in the first 12 h, then further decreased by 50% after 24 h, and finally decreased by 70% after 72 h compared with the new carpet (Table 3, Supplementary Table 5). The concentration of FTOHs after 24 h of the simulated aging process was similar to that of a 3-year-old carpet. The PFCs in the gas phase collected by the XAD-2 sorbent had their concentrations increased over time (Table 3).

In the carpet cleaning test, the concentrations of PFCs in the carpet were reduced by approximately 20% in the first two rounds of cleaning, then further decreased by 50% after five rounds of cleaning, and finally decreased by about 60% after ten rounds of cleaning when compared to the levels in the new carpet (Table 4, Supplementary Table 6). These results indicate that cleaning gradually reduced the residual PFCs derived from the carpet production process. Similar to the simulated aging test, the PFCs in the gas phase collected by the XAD-2 sorbent had their concentrations increased over time (Table 4).

**Table 3:** Carpet accelerated aging on the concentration of perfluorinated compounds.

Time (h)	8:2 FTOH		10:2 FTOH		PFOA	
	Accelerated aging*	Gas-phased*	Accelerated aging*	Gas-phased*	Accelerated aging*	Gas-phased*
12	16,588 (6.53)	1757 (4.31)	9800 (0.88)	3039 (7.40)	32,627 (4.44)	6982 (1.58)
24	9488 (3.08)	19,035 (5.13)	5545 (6.27)	23,741 (4.20)	19,642 (2.00)	54,901 (3.26)
72	4101 (4.62)	22,852 (3.34)	2774 (3.82)	27,874 (1.87)	14,791 (2.28)	64,786 (1.21)

*RSD*: relative standard deviation.

*Concentration (ng/g) (RSD%).

**Table 4:** Carpet cleaning on the concentration of perfluorinated compounds.

Number of cleaning	8:2 FTOH		10:2 FTOH		PFOA	
	Accelerated aging*	Gas-phased*	Accelerated aging*	Gas-phased*	Accelerated aging*	Gas-phased*
2	15,864 (3.66)	1820 (8.57)	9267 (4.42)	3112 (3.63)	30,380 (5.78)	12,139 (6.41)
5	11,492 (6.13)	11,307 (4.82)	7032 (6.23)	12,290 (3.14)	24,045 (5.68)	21,648 (4.33)
10	6433 (1.26)	14,934 (3.50)	3891 (4.52)	17,368 (1.61)	14,142 (5.43)	64,786 (2.72)

*RSD* relative standard deviation.

*Concentration (ng/g) (RSD%).

### Exposure to perfluorooctanoic acid is a risk factor for age-related macular degeneration

**Figure 1:**
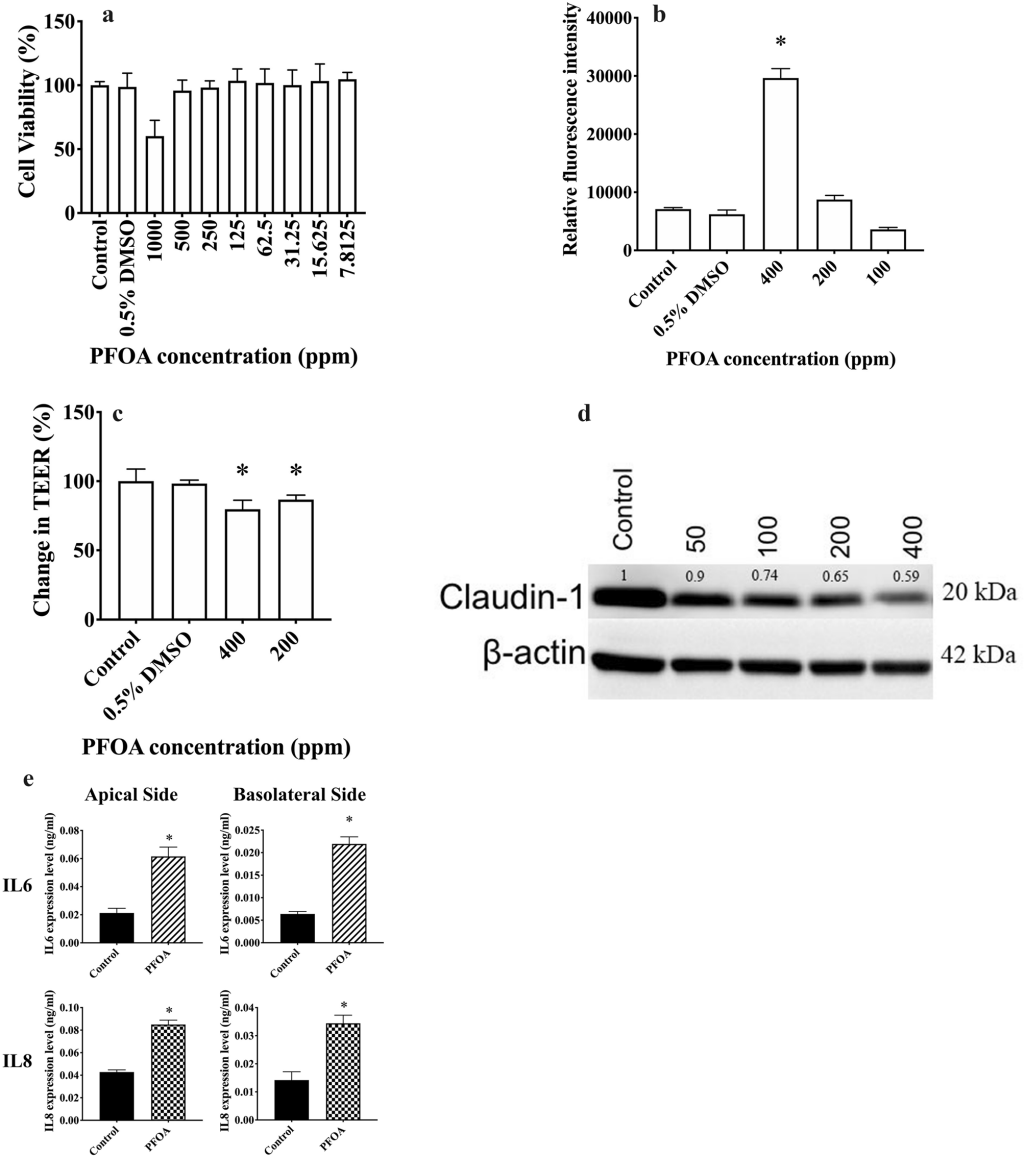
Effects of perfluorooctanoic acid (PFOA) on human corneal cells. (**a**) PFOA showed minimal cytotoxicity against human corneal epithelial cells (HCEpiC) treated with different concentrations of PFOA for 72 h. Cell viability was evaluated using the MTT assay. (**b**) PFOA induced the production of reactive oxygen species (ROS) in HCEpiC treated with different concentrations of PFOA for 6 h. Levels of ROS were determined using 2,7-dichlorofluorescin diacetate (DCFH-DA). Experimental groups were compared using ANOVA (*P* < 0.0001), and Dunnett’s multiple comparisons tests were used for paired comparisons between phosphate-buffered saline (PBS; control) and 0.5% (v/v) DMSO, 400 ppm PFOA, 200 ppm PFOA or 100 ppm PFOA-treated HCEpiC. *P* values below 0.05 were considered to indicate statistical significance. (**c**) PFOA altered tight junctions in HCEpiC. Relative transepithelial electrical resistance (TEER) was measured in HCEpiC incubated with PBS (control), 0.5% DMSO, 400 ppm PFOA, or 200 ppm PFOA for 24 h. Experimental groups were compared using ANOVA (*P* < 0.0001), and Dunnett’s multiple comparisons tests were used for paired comparisons between control and 0.5% DMSO, 400 ppm PFOA or 200 ppm PFOA-treated HCEpiC. *P* values below 0.05 were considered to indicate statistical significance. (**d**) PFOA treatment decreased the expression level of claudin-1. Cells were treated with different concentrations of PFOA for 24 h, and the level of claudin-1 was determined by western blotting. β-actin was used as an endogenous reference protein. The expression levels of claudin-1 were normalized against β-actin and the relative expression levels compared with the control were noted above each lane. (**e**) Levels of IL-6 and IL-8 in HCEpiC. Cells were seeded on transwell inserts with a 0.4-µm pore size incubated with PBS (control) or 400 ppm PFOA for 16 h, and the levels of IL-6 and IL-8 in apical and basolateral compartments were determined using enzyme-linked immunosorbent assays. Unpaired t-test was used to determine significant differences between control and PFOA treated groups. *P* values below 0.05 were considered to indicate statistical significance. Asterisks denote statistical significance.

Cytotoxic effects of PFOA on HCEpiC, the first anatomical barrier of the eye, were evaluated. It was found that PFOA had no significant cytotoxicity against HCEpiC when concentrations up to 500 ppm were used (Fig. 1a). However, PFOA induced ROS production in a dose-dependent manner in HCEpiC (Fig. 1b). Moreover, PFOA-treated HCEpiC had lower transepithelial electrical resistance (TEER) levels (Fig. 1c) and reduced expression of claudin-1, a tight junction protein, when compared to control cells (Fig. 1d). In HCEpiC, PFOA treatment promoted the secretion of IL-6 and IL-8 in both the apical and basolateral media (Fig. 1e).

**Figure 2:**
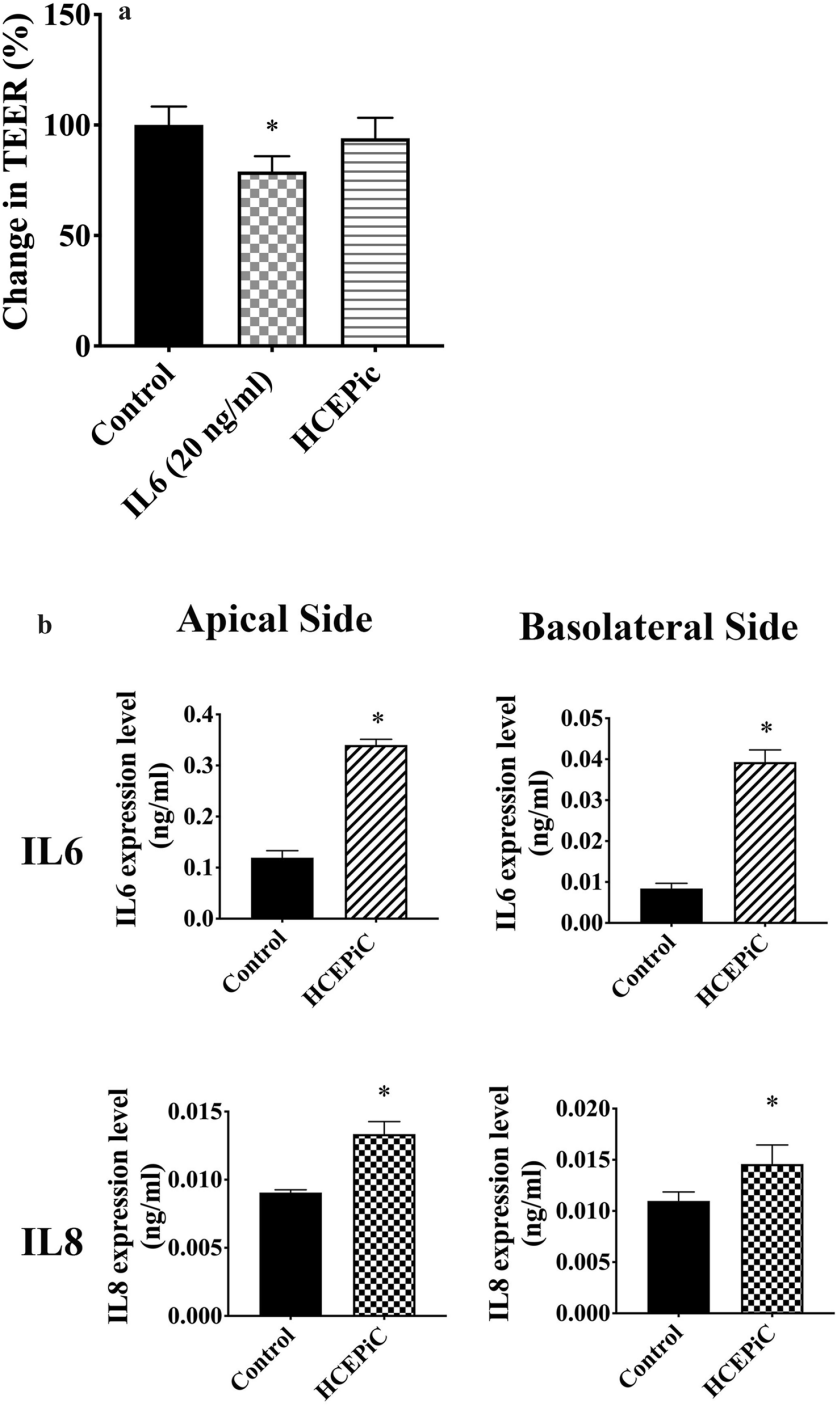
Perfluorooctanoic acid (PFOA) induced the secretion of proinflammatory cytokines by corneal epithelial cells, which affected inflammatory responses of corneal endothelial cells. (**a**) Inflammatory cytokines in the corneal epithelial cells (HCEpiC) basolateral media may alter the tight junction integrity of corneal endothelial cells (HCEC). Relative transepithelial electrical resistance of HCEC incubated with PBS (control), interleukin (IL) 6 (20 ng/mL), or HCEpiC basolateral media for 24 h. Experimental groups were compared using ANOVA (*P* = 0.0014), and Dunnett’s multiple comparisons tests were used for paired comparisons between control and IL-6- or HCEpiC basolateral media-treated HCEC. *P* values below 0.05 were considered to indicate statistical significance. (**b**) Inflammatory cytokines in the corneal epithelial cells (HCEpiC) basolateral media may induce the secretion of inflammatory cytokines by HCEC. Basolateral media of HCEpiC cells treated with PBS (control) or PFOA for 16 h were collected and used to treat HCEC for 16 h. The levels of IL-6 and IL-8 were determined using enzyme-linked immunosorbent assays. Unpaired t-test was used to determine significant differences between control and PFOA treated groups. *P* values below 0.05 were considered to indicate statistical significance. Asterisks denote statistical significance.

Human corneal endothelial cells (HCEC) treated with basolateral media from PFOA-treated HCEpiC did not alter their TEER. However, HCEC treated with 20 ng/mL IL-6 had significantly lower TEER values compared to control cells (Fig. 2a). Moreover, the treatment with basolateral media from PFOA-treated HCEpiC induced the secretion of IL-6 and IL-8 by HCEC in both apical and basolateral media (Fig. 2b).

**Figure 3:**
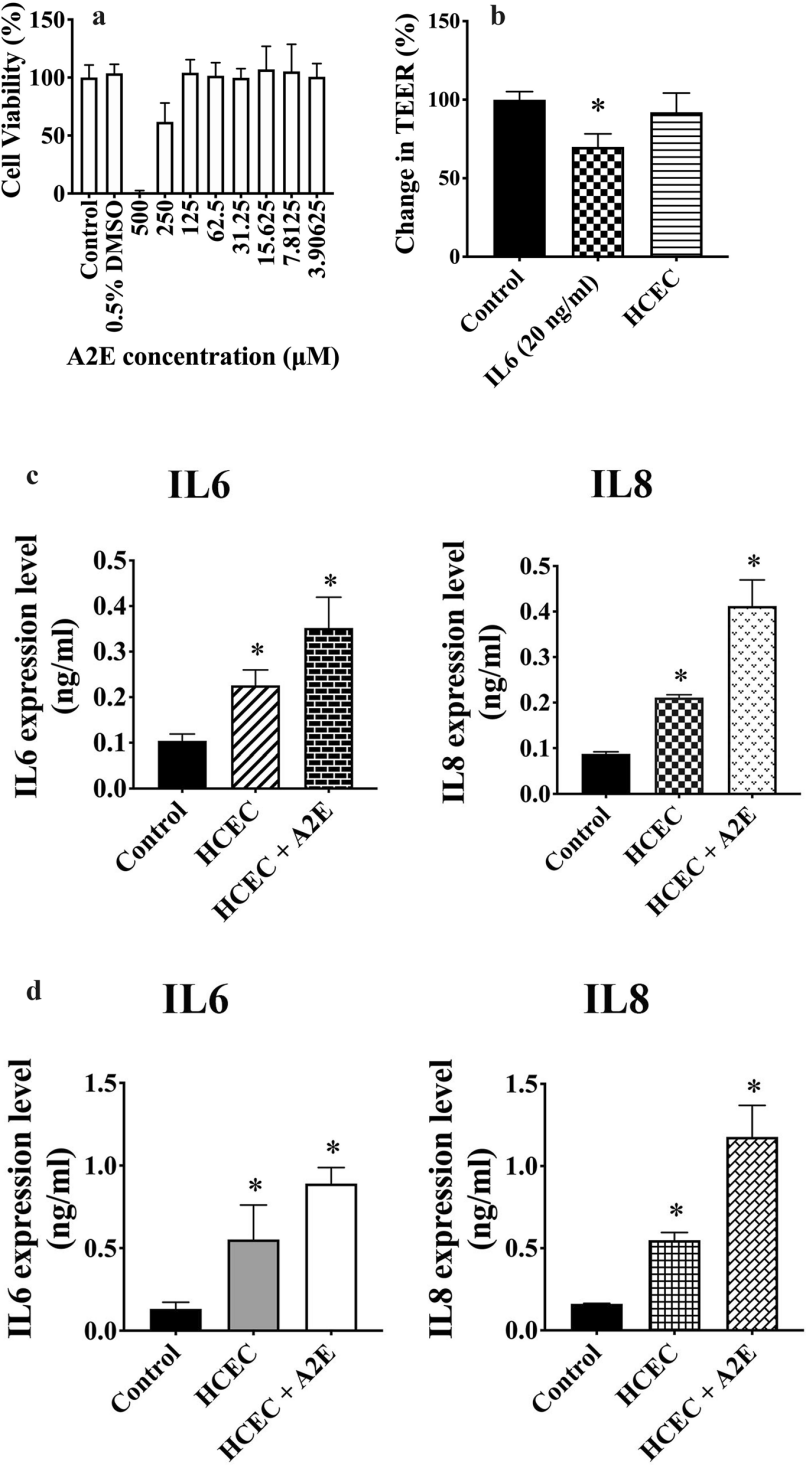
Perfluorooctanoic acid (PFOA) induced inflammation in retinal pigment epithelial cells. (**a**) Cytotoxicity of different concentrations of N-retinylidene-N-retinylethanolamine (A2E) towards retinal pigment epithelial cells (ARPE-19). Cell viability was determined by the MTT assay. (**b**) Inflammatory cytokines in the HCEC basolateral media may disrupt tight junctions in ARPE-19 cells. Relative transepithelial electrical resistance (TEER) of ARPE-19 cells incubated with PBS (control), interleukin (IL) 6 (20 ng/mL), or human corneal endothelial cells (HCEC) basolateral media for 24 h. Experimental groups were compared using ANOVA (*P* = 0.0001), and Dunnett’s multiple comparisons tests were used for paired comparisons between control and IL-6- or HCEC basolateral media-treated ARPE-19 cells. *P* values below 0.05 were considered to indicate statistical significance. (**c**) Basolateral media of HCEC treated with PBS (control) or corneal epithelial cells (HCEpiC) basolateral media for 16 h were collected and used to treat ARPE-19 cells for 16 h. ARPE-19 cells were also treated with 50 μM A2E for 16 h. Levels of IL-6 and IL-8 were determined using enzyme-linked immunosorbent assays. Experimental groups were compared using ANOVA, and Dunnett’s multiple comparisons tests were used for paired comparisons between control and HCEC basolateral media- or HCEC basolateral media + A2E-treated ARPE-19 cells. *P* values below 0.05 were considered to indicate statistical significance. (**d**) Same experiment as in Fig. 3c, using human primary retinal epithelial cells instead of ARPE-19 cells. Asterisks denote statistical significance.

We then assessed whether PFOA could alter the biological function of retinal pigment epithelial cells. First, we determined the cytotoxicity of A2E, one of the major components of lipofuscin that induces oxidative stress and complement system activation to promote AMD, on ARPE-19 cells. Cytotoxic activity against ARPE-19 was observed at 250 μM A2E (Fig. 3a). Thus, we used 50 μM A2E in the following experiments. ARPE-19 cells treated with basolateral media from HCEC (the media from experiments shown in Fig. 2b) did not alter their TEER. However, ARPE-19 cells treated with 20 ng/mL IL-6 had lower values of TEER than control cells (Fig. 3b). Basolateral media from HCEC induced the secretion of IL-6 and IL-8 by ARPE-19 cells (Fig. 3c) and the expression levels of IL-6 and IL-8 were further increased in the presence of A2E (Fig. 3c). The results were very similar when human primary retinal epithelial cells were used instead of ARPE-19 cells. Secretion of IL-6 and IL-8 were significantly induced by HCEC basolateral media, as well as in the presence of A2E (Fig. 3d).

## Discussion

This study provides a potential link between IPM and eye inflammation, which is a risk factor for AMD. We found high concentrations of PFOA in indoor dust samples, especially in places covered with carpets. PFOA was detected in indoor dust samples from 20-year-old carpets as well as from carpets washed ten times. The results confirm the potential for long-term exposure of humans to indoor PFOA.

Fluorotelomer alcohols are volatile PFCs that are more likely to undergo long-range atmospheric transport. The atmospheric lifetime of FTOHs is of approximately 20 days^26^, following which they are degraded to PFOA that is then able to precipitate in remote places^27,28^. This is a possible explanation for the fact that we did not detect FTOHs in the 20-year-old carpet. Thus, the observed high concentration of PFOA in the samples from this carpet might be the result of the degradation of 8:2 FTOH and 10:2 FTOH to PFOA. Residual PFOA has also been found on the surface of nonstick coating on cookware^29^, which allows its release to the air when heated or its mixture with food during cooking. In our experiments, the concentration of PFOA released into the air was significantly lowered after heating (aging) and washing.

In addition to its formation as a product of FTOH degradation, PFOA is one of the most commonly used PFC and can be detected in diverse environments; thus, humans are frequently exposed to PFOA through food and water ingestion. PFOA, with a half-life in serum for 2.3–8.5 years, is not biodegradable and accumulates in living organisms. Exposure to PFOA results in its presence in umbilical cord blood and breast milk, which may potentially affect the health of children^30^. In general, the toxic effects of PFOA include hepatotoxicity, renal toxicity, neurotoxicity, lung toxicity, and general genotoxicity^31^. However, there is no report regarding the potential harmful effects of PFOA against the eyes. Here, we found that PFOA was mainly detected in the PM_2.5_ fraction of indoor dust samples and it could induce inflammatory responses on retinal pigment epithelial cells, which were able to further promote inflammation in retinal cells. Such inflammatory processes may contribute to the development of eye diseases, including AMD.

**Figure 4:**
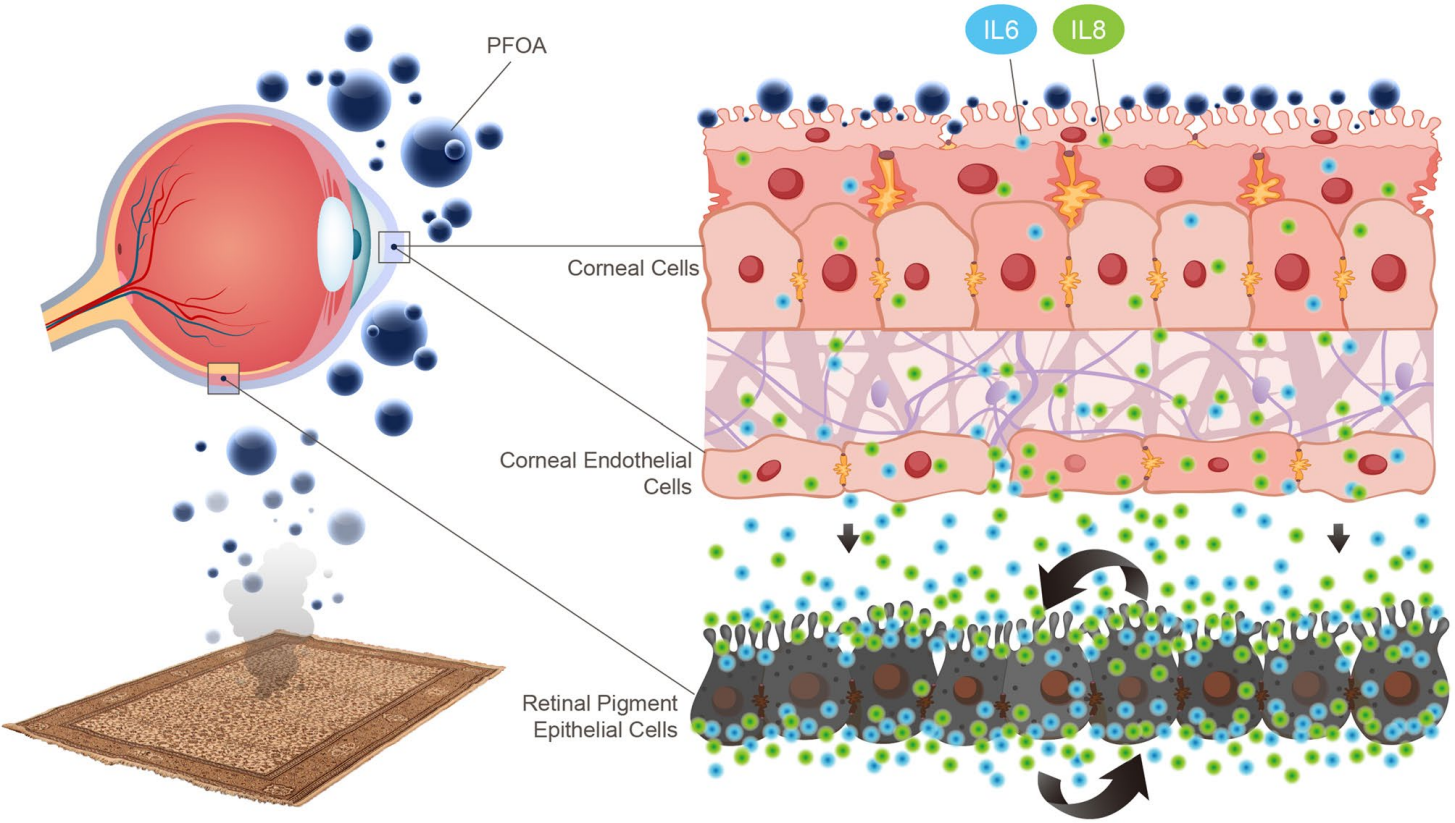
PFOA in the PM 2.5 induced reactive oxygen species and inflammatory reactions across the eye to increase the risk of AMD.

IPM may contact directly with the outermost part of the eye, i.e. the cornea. The outermost layer of the cornea is formed by corneal epithelial cells, which establish tight junctions to prevent foreign material from entering the eye, and its innermost layer is formed by corneal endothelial cells, which can also form tight junctions. Our results suggest that exposure to PFOA alters the tight junctions of corneal epithelial cells and increases the levels of inflammatory cytokines across these epithelial cells. The inflammatory cytokines secreted by the epithelial cells then act on the corneal endothelial cells to alter the tight junctions that thus promote the passage of inflammatory cytokines through the corneal endothelium. These inflammatory cytokines subsequently stimulate the inflammatory cytokine expression and alter the tight junction of retinal pigment epithelial cells, the barrier between the choroid and retina. Such inflammatory reactions, as indicated by the high levels of IL-6 and IL-8 in this study, were further enhanced by A2E treatment, a major component of lipofuscin that is associated with the pathogenesis of AMD. Accordingly, we found that PFOA, a common PFC in PM_2.5_, induced the generation of ROS and disrupted the tight junction integrity in corneal epithelial cells and endothelial cells, which were associated with inflammation in retinal pigment epithelial cells (Fig. 4).

Age-related macular degeneration is characterized by degenerative changes in the outer portion of the retina, RPE, Bruch's membrane, and choriocapillaris^32^. In addition to genetic predispositions, the prevalence of AMD is also associated with lipofuscinogenesis, drusogenesis, local inflammation, and neovascularization^32^. One important indicator of increased risk of AMD (both the atrophic and exudative forms) is the formation of drusen^32^. Drusen are amorphous deposits that accumulate between the RPE basal lamina and the inner collagenous layer of Bruch’s membrane. The tightly-balanced immune system of the retina is disrupted by drusen, which promotes the onset of AMD^33^. Lipofuscin, the main fluorophore in the RPE, is derived from incompletely degraded photoreceptor outer segments. Lipofuscin accumulates in the acidic vacuolar apparatus in RPE cells, which alters the metabolic functions of RPE. When all-transretinal reacts with phosphatidylethanolamine, it generates A2E^34,35^, which promotes the accumulation of lipofuscin in RPE by inhibiting lysosomal proteolysis^36^. Excess A2E in the lysosomes of RPE cells alters the lysosomal pH^37^, which inhibits the activity of lysosomal enzymes and leads to downregulation of further proteolytic degradation of outer segments^38^. Exposure to A2E has been shown to induce the production of inflammatory mediators, such as IL-8, IL-6, and vascular endothelial growth factor-A, in human RPE cells^39^. In addition to its role in inflammatory processes, IL-8 has been shown to promote angiogenesis^40^, which is important in the induction of AMD. IL-6 has been associated with the progression of geographic atrophy secondary to AMD^41^.

In the present manuscript, we did not evaluate the accumulation of PFC in sera but the direct contact of PFC with the cornea may promote the secretion of inflammatory cytokines across the cornea and then increase the inflammatory reactions in the retina, encouraging the development of AMD. It is known that AMD affects more females than males^42^, which may be related to the fact that, traditionally, women are more involved in indoor housekeeping activities^43^, increasing the probability of exposure to PFCs in dust. A more detailed population study should be conducted to understand the correlation between indoor housekeeping activities and the risk of AMD.

Our findings indicate that PFOA contained in indoor dust samples can damage RPE and potentially lead to AMD. Nevertheless, our results from cell-based experiments need to be confirmed by in vivo studies, which represents the next step for research on the role of PFOA in macular degeneration.

## Material and methods

### Indoor dust collection

We collected dust samples from the collection bag of vacuum cleaners^44^ from five different sites: (1) the National Tsing-Hua University (NTHU) Library, HsinChu, Taiwan; (2) the National Chiao-Tung University (NCTU) Library, HsinChu, Taiwan; (3) a construction site; (4) an office of the Department of Biomedical Engineering and Environment Sciences, NTHU, HsinChu, Taiwan; and (5) a 12-square meter home. The hallways and reading areas of the two libraries were carpeted. The carpets in the NTHU library were 20 years old, whereas those in the NCTU library was 3 years old. To assess the effect of natural aging, we also analyzed new carpet samples from the same manufacturer that produced the carpets in the libraries. Samples were sieved to remove particles greater than 50 μm in diameter. We used a Dekati Low Pressure Impactor (DLPI; Dekati, Kangasala, Finland) to determine the gravimetric mass size of the particles.

### Chemical analysis of perfluorinated compounds

#### Fluorotelomer alcohols

Fluorotelomer alcohols (FTOH) were extracted by methanol extraction. After extraction, 50 μL of the supernatant was mixed with 50 μL of internal standard 10:2 FTOH [M + 4] (2500 ng/mL), and samples were analyzed by gas chromatography–mass spectrometry (GC–MS) within 24 h.

#### Perfluorooctanoic acid

The extraction method for analysis of perfluorooctanoic acid (PFOA) was based on procedures described by Fujii et al.^45,46^ and Washington et al.^47^. MilliQ water was added to the dry dust and vortexed until visually homogenized. Then, 200 μL of 2 M NaOH was added and incubated for 30 min at 25 °C. After alkaline pretreatment, acetonitrile was added to yield a 60:40 acetonitrile: H_2_O (v/v) solution. The solution was neutralized by adding 200 μL of 2 M HCl, then sonicated for 60 min and centrifuged at 1500×*g* for 5 min. The suspension was concentrated to 1 mL under a gentle stream of nitrogen gas. Next, 1 mL of a solution containing 0.5 M tetra-butyl-ammonium hydrogen-sulfate (TBAS) and 0.25 M sodium carbonate buffer (pH 10), and 1.2 mL of methyl tert-butyl ether (MTBE) were added to the samples, and incubated for 1 h. Samples were then centrifuged at 1500×*g* for 10 min. The organic layer was collected and then dried under a gentle stream of nitrogen gas. The residue was dissolved in 50 μL of 0.1 M benzyl bromide/acetone solution and 50 μL of internal standard, derivatized at 80 °C for 15 min and then analyzed by GC–MS within 24 h.

The presence and concentration of FTOHs and PFOA in the samples were determined by their retention times and relative abundance of FTOHs or PFOA standards. The standard curve, i.e., the plot of the relative peak areas (analyte/internal standard) versus the analyte concentration, was generated using five concentration levels of standards in triplicate. The coefficients of determination (R^2^) were higher than 0.995 for each calibration. Instrument and method limits for FTOHs and PFOA detection are listed in Supplementary Table 1.

### Carpet accelerated aging test

To simulate the aging of carpets containing PFCs, a piece of new carpet was placed in three-neck round-bottom flasks at 100 °C on silicone oil bath. The flasks were tightly sealed, and air was passed through the flasks at a flow rate of 40 mL/min. Various aging times (12, 24, and 72 h) were studied. Headspace vapors from carpet were collected using sorbent glass tubes, which contained a glass wool to trap aerosols and a two-section sorbent bed (100 mg and 20 mg) of XAD-2 resin to capture vapors. Compounds were extracted from the resin and analyzed for the concentration of FTOHs and PFOA.

### Carpet cleaning test

To simulate the cleaning of carpets containing PFCs, a piece of new carpet and a stir bar were placed in three-neck round-bottom flasks. Carpet detergents (40 mL, 1:50 diluted) were added to the flask, which was heated to 60 °C on silicone oil bath and stirred at 320 rpm for 15 min. After cleaning, the carpet detergents were removed and the flask was washed twice with 40 mL deionized water. The carpet was dried at 50 °C for 12 h prior to extraction. The flasks were tightly sealed, and air was passed through the flasks at a flow rate of 40 mL/min. Headspace vapors from carpet were collected using sorbent glass tubes, which contained glass wool to trap aerosols and a two-section sorbent bed (100 mg and 20 mg) of XAD-2 resin to capture vapors. Compounds were extracted from the resin and analyzed for the concentration of FTOHs and PFOA.

### Cell culture

Human corneal epithelium cells (HCEpiC; passages 3–5) and retinal pigment epithelial cells (HREpiC; passages 3–4) were obtained from ScienCell Research Laboratories (San Diego, CA, USA) and cultured according to the instructions provided by the supplier. Human corneal endothelial cells (HCEC) were obtained from Leibniz Institute DSMZ—German Collection of Microorganisms and Cell Cultures (Braunschweig, Germany). Briefly, HCEC were maintained in human-endothelial-SFM medium with 10 ng/mL fibroblast growth factor-2. Retinal pigment epithelial cells (ARPE-19) were obtained from Bioresource Collection and Research Center, HsinChu, Taiwan, and cultured in Dulbecco’s modified Eagle’s medium/Ham’s F-12 medium (1:1) (90%) supplemented with 10% fetal bovine serum and 1% penicillin/streptomycin. Cells were cultured at 37 °C and 5% CO_2_, and culture medium was renewed every 2–3 days.

### Cell viability assays

To determine the cytotoxicity of PFOA on HCEpiC and that of A2E on ARPE-19, cells were seeded at 5 × 10^3^ cells/well for ARPE-19 and 1 × 10^4^ cells/well for HCEpiC cells in 96-well plates. Cells were treated with their respective culture media containing different concentrations of PFOA or A2E for 72 h at 37 °C. Cell viability was determined using the 3-(4,5-cimethylthiazol-2-yl)-2,5-diphenyl tetrazolium bromide (MTT) assay.

### Transepithelial electrical resistance measurement

To evaluate the effect of PFOA on the tight junction sealing of corneal epithelium and corneal endothelium, 2 × 10^4^ HCEpiC (in 200 μL of medium), 4 × 10^4^ HCEC, or 2 × 10^4^ ARPE-19 cells were plated into Millicell 24-well cell culture inserts (1 μm) (Merck Millipore, Darmstadt, Germany) with 1.3 mL of media in the basolateral site. Media were renewed every 3 days for a total of 14 days. Cells were treated with indicated materials for 24 h. Transepithelial electrical resistance (TEER) was measured using a Millicell ERS-2 Voltohmmeter (Merck, Kenilworth, NJ, USA) according to the manufacturer’s instructions. The electrical resistance was calculated according to the formula:

1 $$ {\text{TEER}}\,(\Omega \,\,{\text{cm}}^{2} ) = \left( {{\text{resistance}}\,{(}\Omega {\text{) - background}}\,{\text{resistance}}\,{(}\Omega {)}} \right) \times {\text{membrane}}\,{\text{area}}\,(0.33\,{\text{cm}}^{2} ) $$

Changes in TEER for each treatment were calculated as:

2 $$ {\text{TEER}}\,{(}\% {)} = ({\text{TEER}}\,{(}\Omega \,\,{\text{cm}}^{2} {)}/{\text{initial}}\,{\text{TEER}}\,{(}\Omega \,{\text{cm}}^{2} {)}) \times 100 $$

### Enzyme-linked immunosorbent assay

Apical and basolateral media were collected to measure the levels of IL-6, and IL-8 using commercially available enzyme-linked immunosorbent assay reagents (Duoset, R&D Systems, Minneapolis, MN, USA). One milliliter of basolateral media was used to treat HREpiC seeded in 6-well plates.

### Detection of reactive oxygen species

HCEpiC were seeded at a concentration of 10,000 cells/well in black 96-well cell culture plates. The cells were then treated with culture medium containing PFOA for 6 h at 37 °C. The generation of ROS was detected using 2,7-dichlorofluorescin diacetate (DCFH-DA). The fluorescence intensities were measured with a fluorescence plate reader (excitation/emission = 480/530 nm).

### Statistical analysis

Experimental groups were compared using ANOVA (*P* < 0.0001), and Dunnett’s multiple comparisons tests were used for paired comparisons between phosphate-buffered saline (PBS; control) and treated groups. *P* values below 0.05 indicate statistical significance.

## Supplementary information

Supplementary Information.

## Data Availability

The data and materials in this manuscript are available from the corresponding authors upon reasonable request.

## Abbreviations

AMD: Age-related macular degeneration
A2E: N-retinylidene-N-retinylethanolamine
FTOH: Fluorotelomer alcohols
GC–MS: Gas chromatography–mass spectrometry
HCEC: Human corneal endothelial cells
HCEpiC: Human corneal epithelial cells
IL: Interleukin
IPM: Indoor particulate matter
PFC: Perfluorinated compounds
PFOA: Perfluorooctanoic acid
ROS: Reactive oxygen species
RPE: Retinal pigment epithelium
TEER: Transepithelial electrical resistance
TNF-α: Tumor necrosis factor-α
