# Supplementary material for: Perfluorooctanoic acid in indoor particulate matter triggers oxidative stress and inflammation in corneal and retinal cells
Source: Sci Rep. 2020 Sep 24;10:15702. doi: 10.1038/s41598-020-72600-8 (PMC7518444; doi:10.1038/s41598-020-72600-8)

Perfluorooctanoic acid in indoor particulate matter triggers oxidative stress and inflammation in corneal and retinal cells

Peng-Tai Tien, Hui-Ju Lin, Yi-Yu Tsai, Yun-Ping Lim, Chih Sheng Chen, Ching-Yao Chang, Chao-Jen Lin, Jamie Jiin-Yi Chen, Shan-Mei Wu, Yuh-Jeen Huang, Lei Wan

Supplement table 1: Quality Assurance for PFCs analysis with GC-MS

Peak Name	8:2FTOH	10:2FTOH	PFOA
Instrument detection limits (ng/mL)	8.31	9.51	549.00
Method detection limits (ng/mL)	9.73	13.73	674.17
Coefficients of calibration curve (R^2)	0.9997	0.9996	0.9994
Recovery% (RSD)	90.25(6.21)	93.26 (7.06)	90.73 (9.58)

Supplement table 2. Weight distribution of different indoor dusts.

		Weight (mg; %)			
Location	Total	$>PM_{10}$	$PM_{10-2.5}$	$PM_{2.5-1}$	$<PM_1$
Library#1	4443.94	4135.8 (93.07)	296.7 (6.68)	11.01 (0.25)	0.36 (0.01)
Library#2	6635.97	6212.3 (93.62)	368.9 (5.56)	53.5 (0.81)	1.35 (0.02)
Decoration	5896.79	5348.3 (90.70)	432.5 (7.33)	111.9 (1.90)	4.05 (0.07)
Office	14315.57	13279.2 (92.76)	852.2 (5.95)	173.9 (1.21)	10.31 (0.07)
House	9955.09	9538.1 (95.81)	362.0 (3.64)	52.4 (0.53)	2.55 (0.03)

* $\text{Percentage}(\%) = \frac{\text{weight(mg)}}{\text{total weight(mg)}} \times 100\%$

Supplementary table 3. Concentrations of perfluorinated compounds for each particle and surface area.

Particle size/ concentration	ng/g	fg/particle	fg/μm^2
**8:2 FTOH**			
**10 ~ 50**	2763	0.0781	0.0000276
**2.5 ~ 10**	14542	0.0036	0.0000308
**1 ~ 2.5**	24796	0.0002	0.0000189
**10:2 FTOH**			
**10 ~ 50**	2763	0.0281	0.000009950
**2.5 ~ 10**	14542	0.0015	0.000013264
**1 ~ 2.5**	24796	0.0001	0.000008001
**PFOA**			
**10 ~ 50**	2763	0.0656	0.0000232
**2.5 ~ 10**	14542	0.0042	0.0000362
**1 ~ 2.5**	24796	0.0003	0.0000205

Supplementary table 4. Concentrations of perfluorinated compounds among different indoor $PM_{2.5-1}$ particulates

Location	Concentration (ng/g), RSD (%)					
	8:2 FTOH		10:2 FTOH		PFOA	
Library#1	24796	3.2	10515	5.5	26882	3.9
Library#2	ND		ND		19304	8.8
Decoration	ND		ND		12752	8.3
Office	ND		ND		15716	5.4
House	ND		ND		17453	8.2

RSD: relative standard deviation

ND: not detectable

Supplementary table 5. Concentration change rate of perfluorinated compounds in carpet (ng g^{-1}/h) during accelerated aging

Compound	Concentration change rate of carpet (ng g^{-1}/hr)		
	12 hr	24 hr	72 hr
8:2 FTOH	402.3	497.0	238.2
10:2 FTOH	166.0	260.3	125.3
PFOA	402.6	742.3	314.8

Supplementary table 6. Concentration change rate of perfluorinated compounds in carpet (ng g^{-1}/h) during carpet cleaning

Compound	Concentration change rate of carpet (ng g^{-1}/hr)		
	12 hr	24 hr	72 hr
8:2 FTOH	2775.7	1457.3	1012.0
10:2 FTOH	1262.5	744.9	628.3
PFOA	3538.9	2111.7	1980.7

Claudin-1

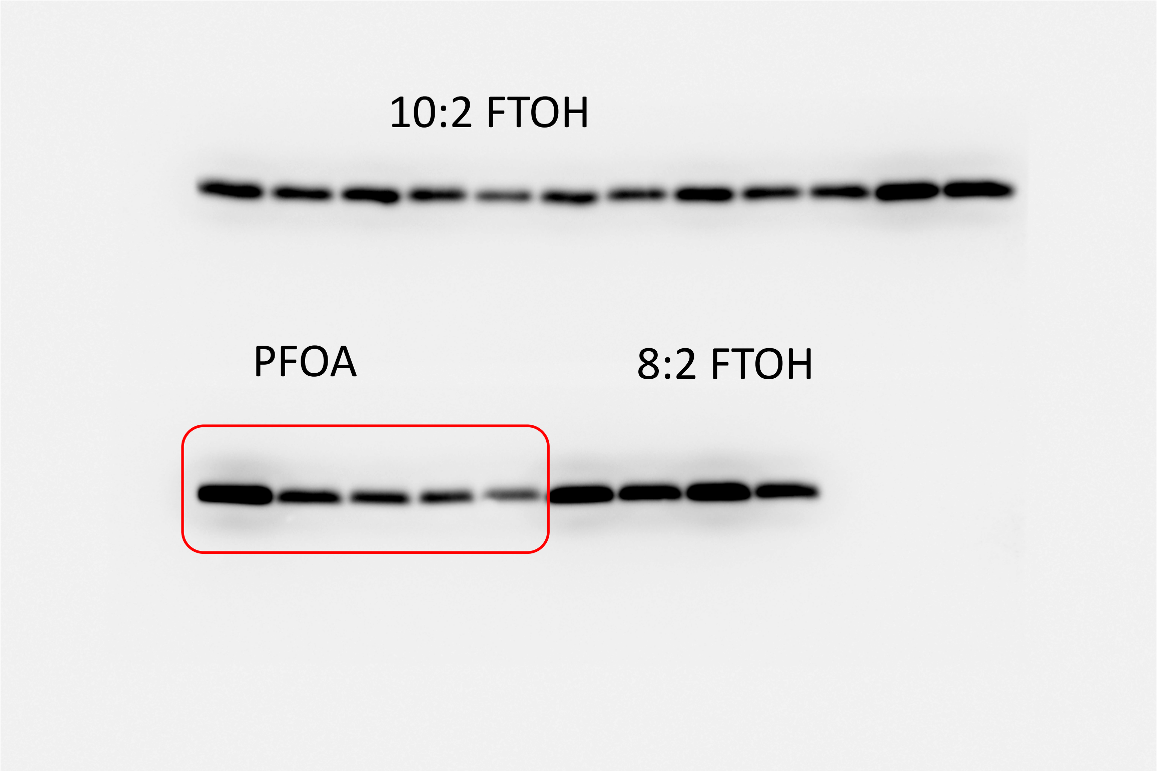

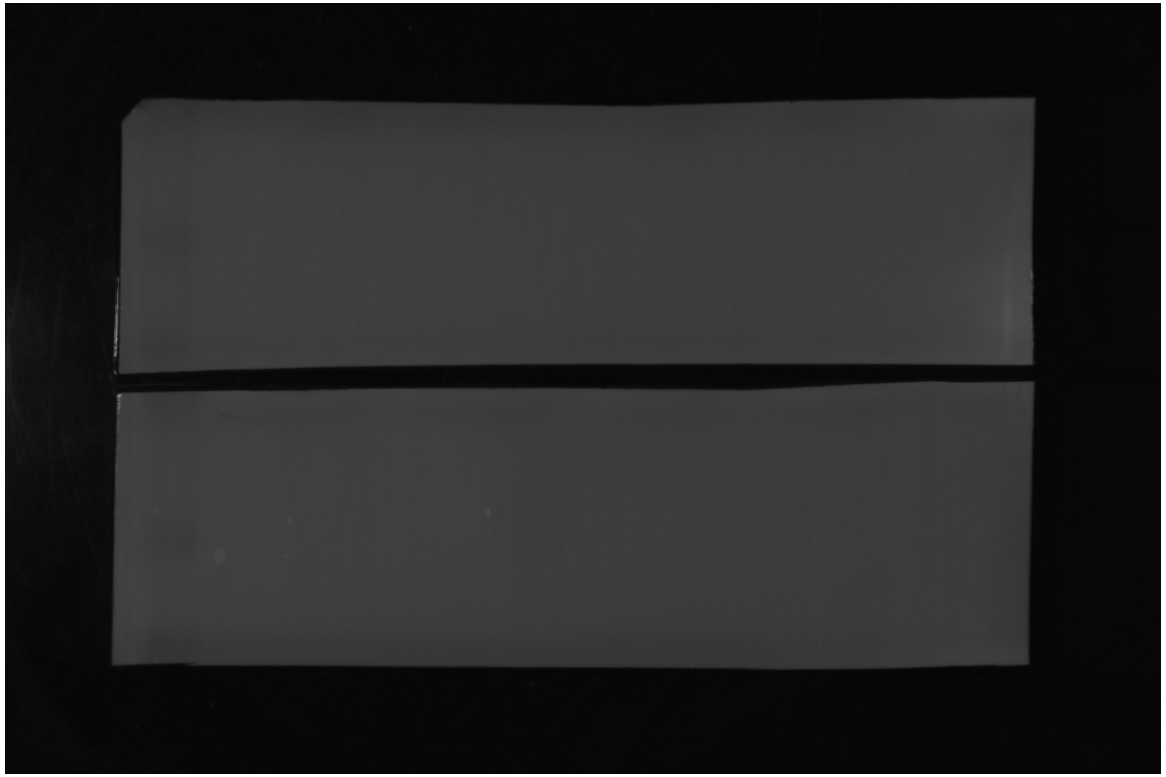

β-actin

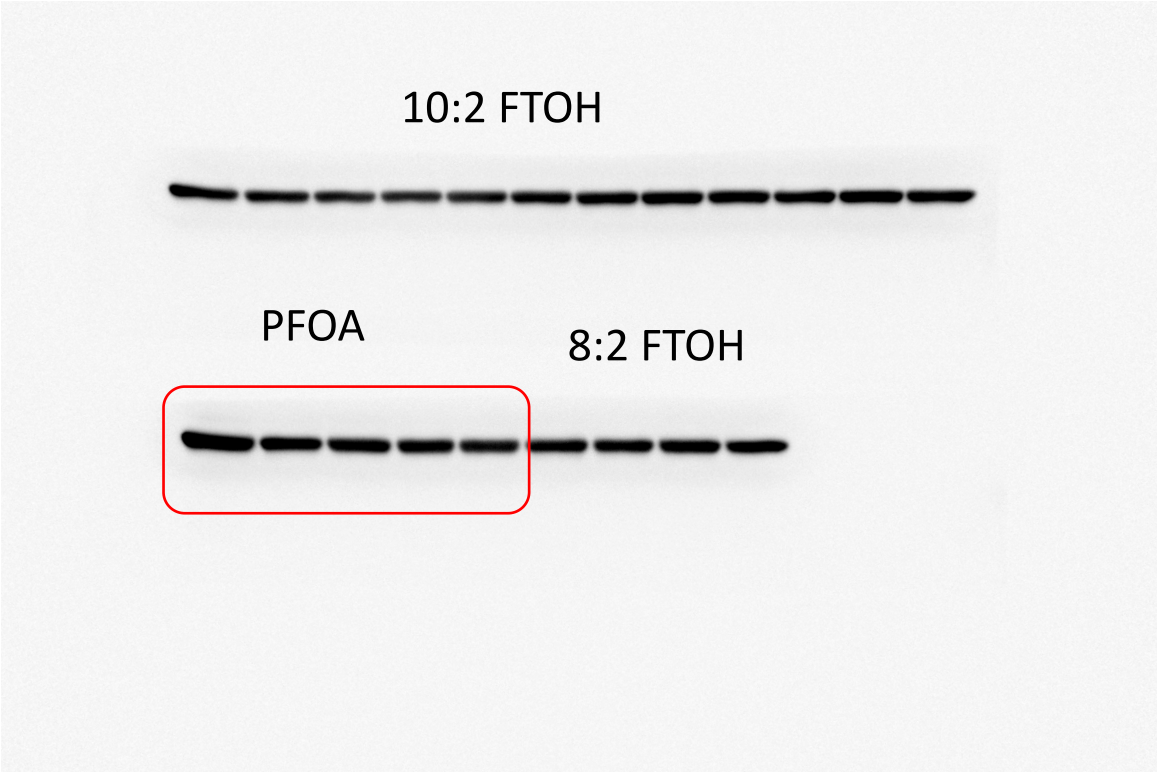

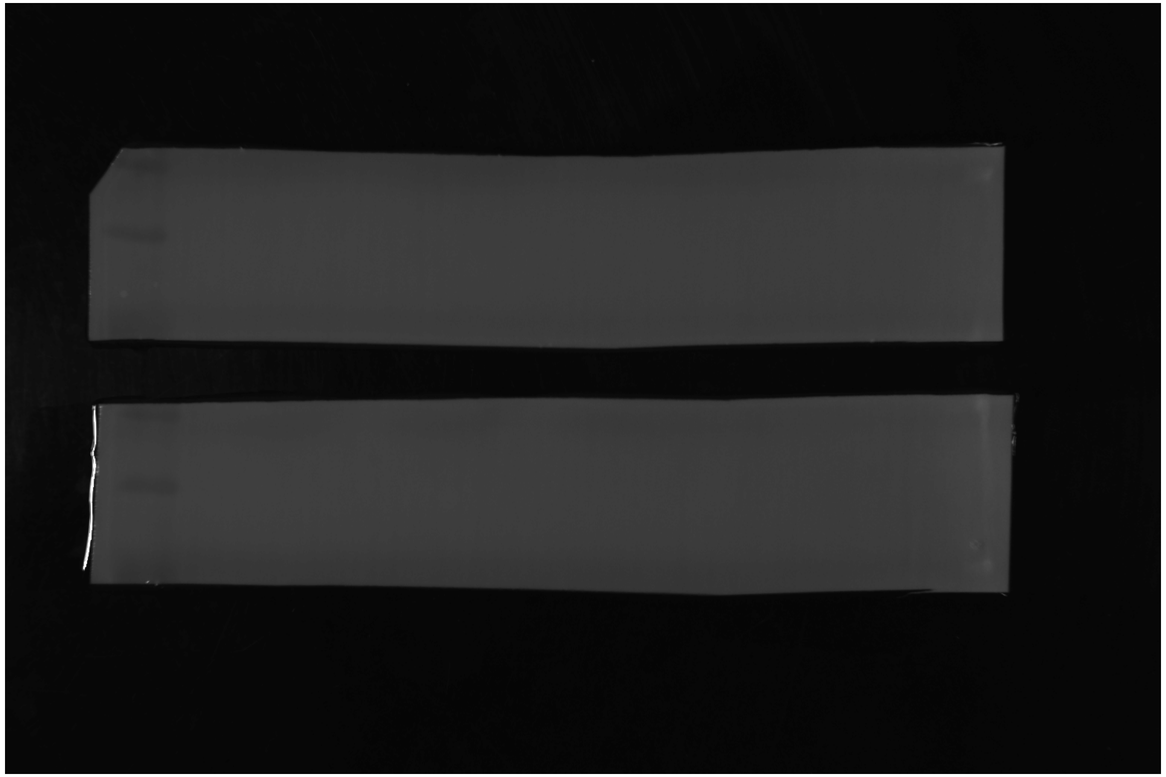

Supplement: Supplementary file 1 — Supplementary Information. [file 41598_2020_72600_MOESM1_ESM.pdf]
